# Comparing Artificial Intelligence–Generated and Clinician-Created Personalized Self-Management Guidance for Patients With Knee Osteoarthritis: Blinded Observational Study

**DOI:** 10.2196/67830

**Published:** 2025-05-07

**Authors:** Kai Du, Ao Li, Qi-Heng Zuo, Chen-Yu Zhang, Ren Guo, Ping Chen, Wei-Shuai Du, Shu-Ming Li

**Affiliations:** 1 Beijing University of Chinese Medicine Beijing China; 2 Beijing Hospital of Traditional Chinese Medicine Beijing China

**Keywords:** artificial intelligence in health care, large language models, knee osteoarthritis, self-management, personalized medicine, patient education, artificial intelligence, AI-generated, knee, osteoarthritis, observational study, GPT-4, ChatGPT, LLMs, orthopedics

## Abstract

**Background:**

Knee osteoarthritis is a prevalent, chronic musculoskeletal disorder that impairs mobility and quality of life. Personalized patient education aims to improve self-management and adherence; yet, its delivery is often limited by time constraints, clinician workload, and the heterogeneity of patient needs. Recent advances in large language models offer potential solutions. GPT-4 (OpenAI), distinguished by its long-context reasoning and adoption in clinical artificial intelligence research, emerged as a leading candidate for personalized health communication. However, its application in generating condition-specific educational guidance remains underexplored, and concerns about misinformation, personalization limits, and ethical oversight remain.

**Objective:**

We evaluated GPT-4’s ability to generate individualized self-management guidance for patients with knee osteoarthritis in comparison with clinician-created content.

**Methods:**

This 2-phase, double-blind, observational study used data from 50 patients previously enrolled in a registered randomized trial. In phase 1, 2 orthopedic clinicians each generated personalized education materials for 25 patient profiles using anonymized clinical data, including history, symptoms, and lifestyle. In phase 2, the same datasets were processed by GPT-4 using standardized prompts. All content was anonymized and evaluated by 2 independent, blinded clinical experts using validated scoring systems. Evaluation criteria included efficiency, readability (Flesch-Kincaid, Gunning Fog, Coleman-Liau, and Simple Measure of Gobbledygook), accuracy, personalization, and comprehensiveness and safety. Disagreements between reviewers were resolved through consensus or third-party adjudication.

**Results:**

GPT-4 outperformed clinicians in content generation speed (530.03 vs 37.29 words per min, *P*<.001). Readability was better on the Flesch-Kincaid (mean 11.56, SD 1.08 vs mean 12.67 SD 0.95), Gunning Fog (mean 12.47, SD 1.36 vs mean 14.56, SD 0.93), and Simple Measure of Gobbledygook (mean 13.33, SD 1.00 vs mean 13.81 SD 0.69) indices (all *P*<.001), though GPT-4 scored slightly higher on the Coleman-Liau Index (mean 15.90, SD 1.03 vs mean 15.15, SD 0.91). GPT-4 also outperformed clinicians in accuracy (mean 5.31, SD 1.73 vs mean 4.76, SD 1.10; *P*=.05, personalization (mean 54.32, SD 6.21 vs mean 33.20, SD 5.40; *P*<.001), comprehensiveness (mean 51.74, SD 6.47 vs mean 35.26, SD 6.66; *P*<.001), and safety (median 61, IQR 58-66 vs median 50, IQR 47-55.25; *P*<.001).

**Conclusions:**

GPT-4 could generate personalized self-management guidance for knee osteoarthritis with greater efficiency, accuracy, personalization, comprehensiveness, and safety than clinician-generated content, as assessed using standardized, guideline-aligned evaluation frameworks. These findings underscore the potential of large language models to support scalable, high-quality patient education in chronic disease management. The observed lexical complexity suggests the need to refine outputs for populations with limited health literacy. As an exploratory, single-center study, these results warrant confirmation in larger, multicenter cohorts with diverse demographic profiles. Future implementation should be guided by ethical and operational safeguards, including data privacy, transparency, and the delineation of clinical responsibility. Hybrid models integrating artificial intelligence–generated content with clinician oversight may offer a pragmatic path forward.

## Introduction

Knee osteoarthritis (OA) is a prevalent, chronic, degenerative joint disorder that imposes a substantial burden on patients’ quality of life and overall well-being [1]. Epidemiological studies have revealed that knee osteoarthritis affects hundreds of millions of individuals worldwide, with its prevalence increasing with age [2]. In addition to age, other major risk factors for knee osteoarthritis include obesity, history of joint injury or surgery, and excessive joint usage due to occupational or athletic activities [3]. Knee osteoarthritis causes pain and functional limitations for individual patients. In addition, it places a heavy burden on society and the economy, leading to decreased work productivity and increased health care costs [4].

Although knee osteoarthritis remains incurable, appropriate treatment and management can effectively alleviate symptoms, slow disease progression, and improve prognosis. Patient education and self-management guidance play a pivotal role in knee osteoarthritis management [5,6]. By providing patients with comprehensive education and personalized guidance on disease knowledge, risk factors, treatment options, and self-management strategies, health care providers can enhance patients’ understanding of their condition, strengthen their self-efficacy, and ultimately improve treatment adherence and health outcomes [7,8].

However, in real-world clinical practice, the imbalance in patient-provider ratio and limited time and resources often constrain the breadth and depth of patient education. Physicians frequently struggle to offer adequate, individualized guidance to each patient, leading to inconsistent education quality [9]. This issue is particularly prominent in knee osteoarthritis management, as the disease course is lengthy, and the patient population is highly heterogeneous [10]. Patients with knee osteoarthritis vary considerably in terms of disease severity, symptom presentation, comorbidities, lifestyle factors, and health literacy levels, necessitating more personalized guidance [11]. Yet, in current clinical practice, the education and guidance received by patients with knee osteoarthritis often remain superficial and one-size-fits-all [12]. This highlights the pressing need for innovative patient education models that can enhance the efficiency and quality of guidance while reducing the burden on clinicians.

The advancement of artificial intelligence (AI) technologies, particularly the breakthroughs in natural language processing, offers a potential solution to the challenges. In recent years, large language models (LLMs) based on transformer architectures, such as ChatGPT (OpenAI), Gemini (Google DeepMind), and Claude (Anthropic), have emerged as promising tools in the medical domain [13]. These LLMs can acquire human-like language understanding and generation capabilities through training on vast amounts of text data, demonstrating remarkable performance in tasks such as medical literature analysis, clinical decision support, information extraction, disease monitoring, and diagnosis, ultimately enhancing efficiency and accuracy in health care processes [14-20]. The powerful information extraction, knowledge integration, and content generation abilities of LLMs hold promise for assisting physicians in delivering personalized patient education, thereby improving education quality and efficiency while reducing clinicians’ workload [21,22].

Despite the promising potential of LLMs in health care, their application in guiding self-management for patients with knee osteoarthritis remains largely unexplored [23-25]. Although LLMs have demonstrated impressive capabilities in generating medical content, most studies to date have primarily focused on general applications in health care, such as improving medical documentation or supporting clinical decision-making, rather than on personalized patient education for specific conditions like knee osteoarthritis [18,19]. Given the unique challenges patients with knee osteoarthritis face in managing their condition, targeted research is urgently needed to explore how LLMs can be applied effectively in this area, identifying both their potential benefits and limitations.

Among available LLMs, GPT-4 was chosen for this study due to its exceptional performance in natural language understanding, long-context reasoning, and medically oriented text generation. It is currently the most widely studied and cited model in clinical AI research, offering a robust benchmark for evaluating feasibility and quality [26]. While other models such as Claude and Gemini show promise, GPT-4 remains the most representative and accessible high-performance model for academic and clinical exploration. Therefore, it serves as a suitable candidate for this exploratory comparison of AI-generated versus clinician-provided personalized patient education.

Nevertheless, the integration of LLMs into clinical care also raises important concerns. Studies have highlighted risks such as the generation of inaccurate or misleading information (hallucinations), insufficient contextual understanding, and a lack of true personalization despite fluent language output [27]. These limitations may lead to inappropriate guidance, particularly when nuanced clinical judgment or cultural sensitivity is required. Furthermore, ethical issues such as patient data privacy, model transparency, algorithmic bias, and accountability in clinical decision-making remain unresolved. Without proper oversight, the application of LLMs in patient education could inadvertently compromise care quality or equity [28]. Therefore, a rigorous, evidence-based evaluation of LLM-generated content is essential before such tools can be safely integrated into real-world health care settings.

This study seeks to explore the potential of LLMs, specifically GPT-4, in generating personalized self-management guidance for patients with knee OA, and to compare its effectiveness with that of physician-provided guidance. By assessing key factors (efficiency, readability, accuracy, comprehensiveness, personality, and safety), this research aims to provide a novel approach to enhancing patient education in knee OA. The study’s findings are expected to shed light on the feasibility of integrating AI-based models into clinical practice, potentially paving the way for more tailored and efficient patient education solutions across various chronic diseases.

## Methods

### Study Design

**Figure 1:**
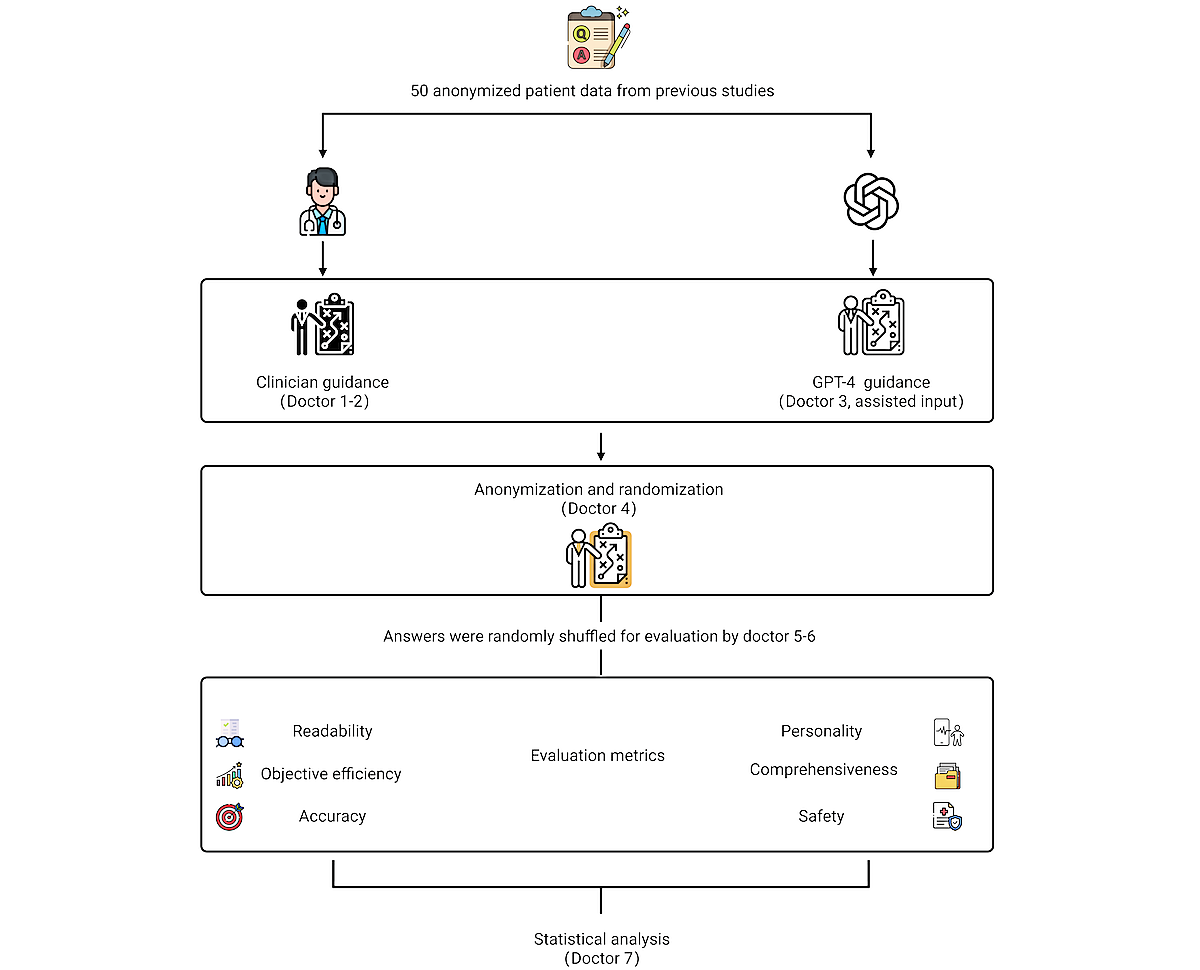
Flowchart of overall study design.

This retrospective study, conducted at the Pain Department of Beijing Hospital of Traditional Chinese Medicine (affiliated with Capital Medical University), used a 2-phase, blinded, observational approach to compare the effectiveness of LLMs, specifically OpenAI’s ChatGPT-4, in generating personalized patient education content for patients with knee osteoarthritis with against content manually created by clinicians. The study used anonymized patient data from a previous trial, which included detailed information about patients’ conditions, symptoms, and medical history. This study’s flowchart is shown in Figure 1.

In the first phase, from August 1 to August 15, 2024, Doctor 1 (RG) and Doctor 2 (SML; both orthopedic specialists with a minimum of 10 years’ experience in knee osteoarthritis management) generated personalized patient education materials. These specialists were selected based on their comparable educational background, clinical experience, and familiarity with the latest knee osteoarthritis management guidelines to minimize individual variability. The anonymized patient data from the previous trial were randomly divided into 2 sets, each containing 25 unique patient profiles. These datasets were then randomly assigned to the 2 specialists, ensuring that each specialist received 25 patient profiles to work with. The specialists were provided with comprehensive information about patients’ current condition, symptomatology, medical history, and knee OA–relevant lifestyle factors based on the assigned patient profiles. Each specialist was given 15 days to create personalized education content for their assigned patients. Content creation occurred within this standardized timeframe to ensure consistency across all manually generated materials, which served as the control group for subsequent analysis.

In the second phase, from August 16 to August 20, 2024, the same 50 anonymized patient datasets used in the first phase were input into GPT-4, an advanced LLM. Doctor 3 [AL] was responsible for assisting with the data input, ensuring consistency in the interaction with GPT-4. To maintain consistency and minimize potential bias, all GPT-4 interactions were conducted by a single researcher within a standardized timeframe, using default parameter settings. Each patient dataset was used to initiate a new GPT-4 conversation session, ensuring the independence of responses. The researcher began each session with a standardized set of instructions: “You are an orthopedic surgeon with extensive experience treating osteoarthritis of the knee.” This was followed by the prompt,

“(1) Analyze the patient’s condition carefully, step by step. (2) Make full use of all the data provided to create educational content and self-management guidance for patients to help them better understand and manage their health. (3) Avoid including specific patient details in the response.”

To mitigate bias, both manually created and LLM-generated content underwent rigorous anonymization and randomization by Doctor 4 (QHZ) before evaluation. A separate cohort of 2 clinical experts, Doctor 5 (WSD) and Doctor 6 (PC), each with a minimum of 5 years’ experience in knee osteoarthritis management and uninvolved in the initial content creation assessed all educational materials. These evaluators were blinded to the content origin, ensuring objective assessment. The study encompassed 100 pieces of educational content (50 GPT-4–generated and 50 human expert–generated), corresponding to 50 unique patient profiles. To address potential discrepancies, if the 2 evaluators’ scores differed by more than 10 points on any dimension, they discussed to reach consensus; if unresolved, a third independent clinician provided an additional assessment, and the median of the three scores was used as the final score.

### Outcomes

#### Objective Efficiency

Objective efficiency was evaluated to compare the productivity of GPT-4 and human experts in generating patient education content. This metric was quantified by calculating the words per minute (WPM) produced during the content creation process [29]. Both GPT-4 and the human experts were timed during their respective content generation tasks. The total word count of the produced content was divided by the time taken (in min) to generate the WPM figure. This measure provides an objective assessment of the speed and efficiency of content production, allowing for a direct comparison between AI-generated and human expert–created materials. However, it should be noted that WPM only reflects the speed of content generation and does not account for the quality or accuracy of the produced content.

#### Readability Evaluation

Readability is a crucial factor in determining the effectiveness of patient education materials, as it directly impacts patients’ ability to understand and engage with the provided information. The readability of patient education materials was assessed using the Readable tool. This instrument uses 4 established readability metrics: the Flesch-Kincaid Grade Level, Gunning Fog Index, Coleman-Liau Index, and Simple Measure of Gobbledygook (SMOG) Index.

The Flesch-Kincaid Grade Level is a widely used readability formula that assesses the approximate reading grade level of a text, based on average sentence length and word complexity [30]. It provides a score that corresponds to a US grade level, indicating the level of education needed to understand the text. Lower scores suggest that the text is easier to read and comprehend.

The Gunning Fog Index is another readability formula that estimates the years of formal education needed to understand a piece of writing on the first reading [31]. It considers the average sentence length and the number of complex words (defined as those with 3 or more syllables). A lower Gunning Fog score indicates that the text is more accessible to a wider audience.

The Coleman-Liau Index is a readability measure that relies on characters instead of syllables per word and sentence length [32]. It calculates the approximate US grade level needed to comprehend a text, with lower scores indicating greater ease of understanding.

SMOG Index is a readability formula that estimates the years of education needed to fully understand a piece of writing [33]. It is particularly well-suited for evaluating health-related materials, as it was developed using a sample of 30-sentence health education materials. Lower SMOG scores suggest that the text is more accessible to readers with less education.

This comparative analysis aims to evaluate the relative accessibility of patient education materials from different sources, as higher readability may lead to better patient understanding, engagement, and ultimately, more effective knee osteoarthritis management.

### Accuracy Evaluation

To evaluate the accuracy of educational content on knee osteoarthritis treatment, we developed a weighted consensus approach designed to ensure alignment with current evidence-based recommendations. This novel methodology synthesizes treatment guidelines from leading professional societies, including the American College of Rheumatology (ACR), Osteoarthritis Research Society International (OARSI), European Society for Clinical and Economic Aspects of Osteoporosis, Osteoarthritis and Musculoskeletal Diseases (ESCEO), American Academy of Orthopaedic Surgeons (AAOS), and National Institute for Health and Care Excellence (NICE) [34-38]. Drawing inspiration from established multisource consensus methods, such as the Delphi technique [39], our approach assigns scores and weights based on the level of agreement across these societies, providing a structured and quantitative assessment of guideline concordance (Multimedia Appendix 1).

Treatment recommendations were categorized and assigned scores based on their endorsement levels: strongly recommended (+2), conditionally recommended (+1), inconclusive (0), conditionally recommended against (–1), and strongly recommended against (–2). This categorization and scoring system allows for a nuanced assessment of the strength of each recommendation. Weights were assigned to each recommendation to reflect the level of agreement among the societies. High consensus (agreement among ≥4 societies) received a weight of 1, moderate consensus (3 societies) of 0.75, low consensus (2 societies) of 0.5, and minimal consensus (1 society) of 0.25. The weighted score for each treatment recommendation was calculated by multiplying the recommendation score by the consensus weight, thereby giving more importance to recommendations with higher levels of agreement among societies.

A comprehensive table was constructed to summarize the consensus levels, weights, and weighted scores for various knee osteoarthritis treatments. The overall accuracy score for the educational content was determined by summing the weighted scores for all treatments mentioned. Higher scores indicated better alignment with consensus guidelines, while lower or negative scores highlighted areas for potential revision. This approach provides a rigorous and objective methodology for evaluating the accuracy of educational content, enhancing its reliability and clinical relevance. A detailed description of the methodology and calculations can be found in Multimedia Appendix 1.

### Personality Evaluation

Personalized content is essential for effective patient education and engagement in knee osteoarthritis management [40]. To assess the degree of personalization in the educational content for knee osteoarthritis treatment, we developed a systematic scoring method tailored to ensure that the content reflects patients’ specific characteristics, symptoms, disease stage, lifestyle habits, and medical history. This novel approach, inspired by principles of personalized medicine and existing frameworks for patient-centered education, comprises 3 key dimensions: relevance (30 points), individual relevance (40 points), and detail level (30 points) [41].

The relevance dimension scored from 0 to 30 points, focused on how well the content addressed the patient’s symptoms and disease stage. The individual relevance dimension scored from 0 to 40 points, evaluated the customization of the content to the patient’s personal characteristics, lifestyle habits, and medical history. The detail level scored from 0 to 30 points, assessed the depth of information provided and the practicality of the suggestions.

Each of these dimensions was scored according to specific criteria, and the total score for personality was calculated out of 100 points. This comprehensive and objective evaluation method ensures that the content is tailored to the unique needs of each patient, potentially enhancing treatment adherence and outcomes. A detailed description of the personality scoring system can be found in Multimedia Appendix 2.

### Comprehensiveness Evaluation

To ensure the comprehensiveness of patient education content generated by both clinicians and LLMs, we developed a systematic evaluation method (Multimedia Appendix 3). This approach assesses whether the content adequately covers all essential topics related to patient care, providing crucial information for effective disease management. The evaluation focuses on 5 key categories: medication treatment, nonmedication treatment, lifestyle advice, psychological support, and disease management. Each category is scored on a scale from 0 to 20 points, with specific criteria for each score range (eg, 17-20 points for complete coverage and 13-16 points for mostly covered). The total possible score is 100 points. This scoring system guarantees that all critical aspects of patient care are thoroughly addressed, offering a well-rounded and informative framework for patient education. By ensuring the comprehensiveness of the content, this method serves as a reliable tool for supporting patient care and improving health outcomes.

### Safety Evaluation

To ensure the safety of patient education content generated by both human experts and LLMs for knee osteoarthritis management, we developed a systematic safety evaluation method (Multimedia Appendix 4). This novel approach, informed by established principles of patient safety in medical education and health intervention risk assessment, evaluates content across 5 critical domains, such as medication treatment, nonmedication treatment, lifestyle advice, psychological support, and disease management [42]. Each domain is assessed for potential risks or harm, such as drug interactions, contraindications, or inappropriate lifestyle recommendations using a structured scoring system. In this system, each domain is rated from 0 to 20 points (eg, 16-20 points for very safe and 0 points for unsafe), yielding a total safety score out of 100 points.

This comprehensive and objective method ensures that all recommendations are free from significant risks, offering a reliable framework to assess the safety and quality of patient education materials. By prioritizing patient safety, our evaluation approach supports the development of high-quality content that promotes optimal care and minimizes harm.

### Statistical Analysis

Statistical analyses were conducted by Doctor 7 (CYZ) using SPSS software (version 27.0, IBM Corp). Continuous variables with normal distribution were presented as means and SDs; nonnormal variables were reported as medians and IQR. The means of 2 continuous normally distributed variables were compared by paired-samples *t* test, while the medians of 2 continuous nonnormally distributed variables were compared by Wilcoxon signed-rank test. The value of *P*<.05 was considered significant.

### Ethical Considerations

Participants in this study were sourced from our earlier research project, “Acupotomy for Knee Osteoarthritis: A Randomized Controlled Trial,” conducted at Beijing Hospital of Traditional Chinese Medicine, Capital Medical University [43]. The original trial was registered with the Chinese Clinical Trial Registry (ChiCTR2100043005) on February 4, 2021, and received ethical approval from the hospital’s Medical Ethical Review Committee (number 2020BL02-057-02). All participants provided written informed consent, which included a provision allowing for the secondary use of their data in future research. To protect participants’ privacy, all personal identifiers had been removed from the dataset before our analysis. All study procedures were conducted in accordance with the Declaration of Helsinki.

## Results

### Objective Efficiency Results

**Figure 2:**
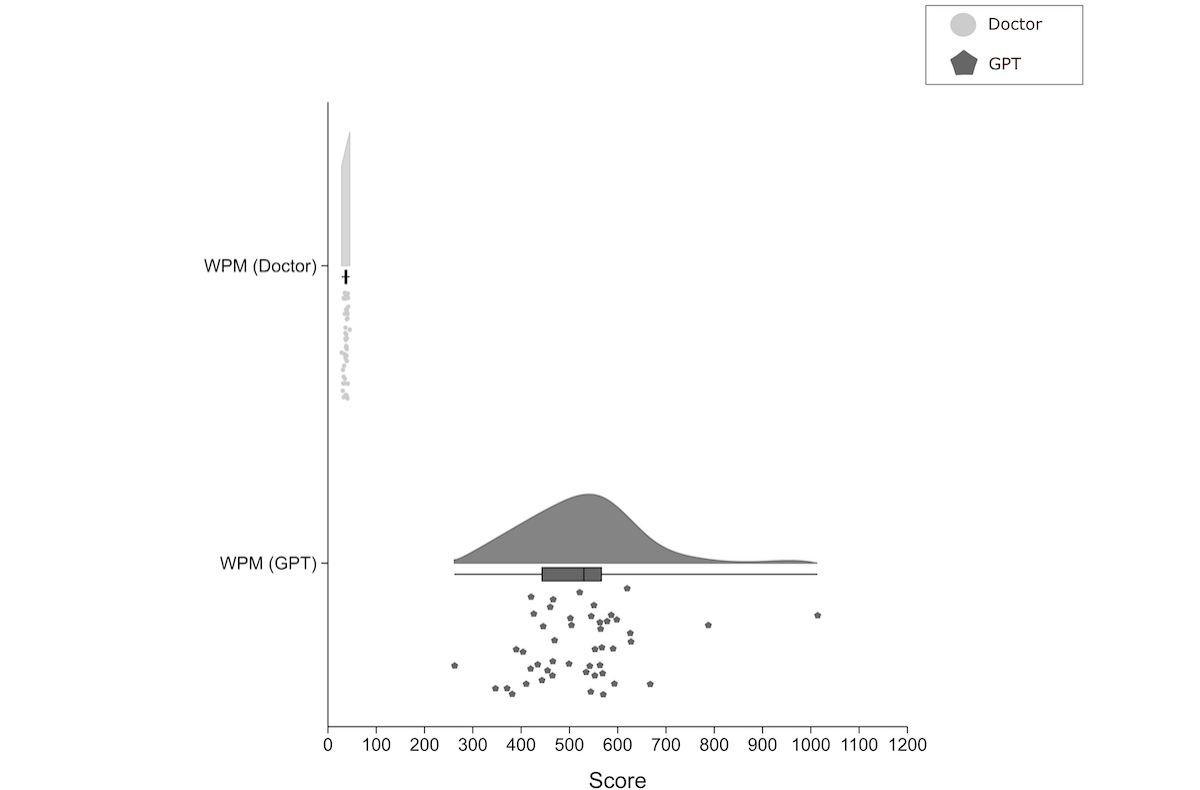
Comparison of objective efficiency between doctors and GPT-4. Raincloud plot showing the distribution of words per minute for patient education generated by doctors and GPT-4. The plot includes a density curve, box plot, and scatter plot of individual data points. Doctors have a median of 37.29 (IQR 35.05-38.99) words per minute, while GPT-4 has a median of 530.03 (IQR 440.39-567.42) words per minute. WPM: words per minute.

Objective efficiency, assessed by WPM, revealed a disparity in content generation speed between clinicians and GPT-4 (Figure 2). Clinicians produced a median of 37.29 (IQR 35.05-38.99) WPM, whereas GPT-4 achieved a median output of 530.03 (IQR 440.39-567.42) WPM, approximately 14 times faster than clinicians. This difference in efficiency was statistically significant (*P*<.001).

### Readability Evaluation Results

**Figure 3:**
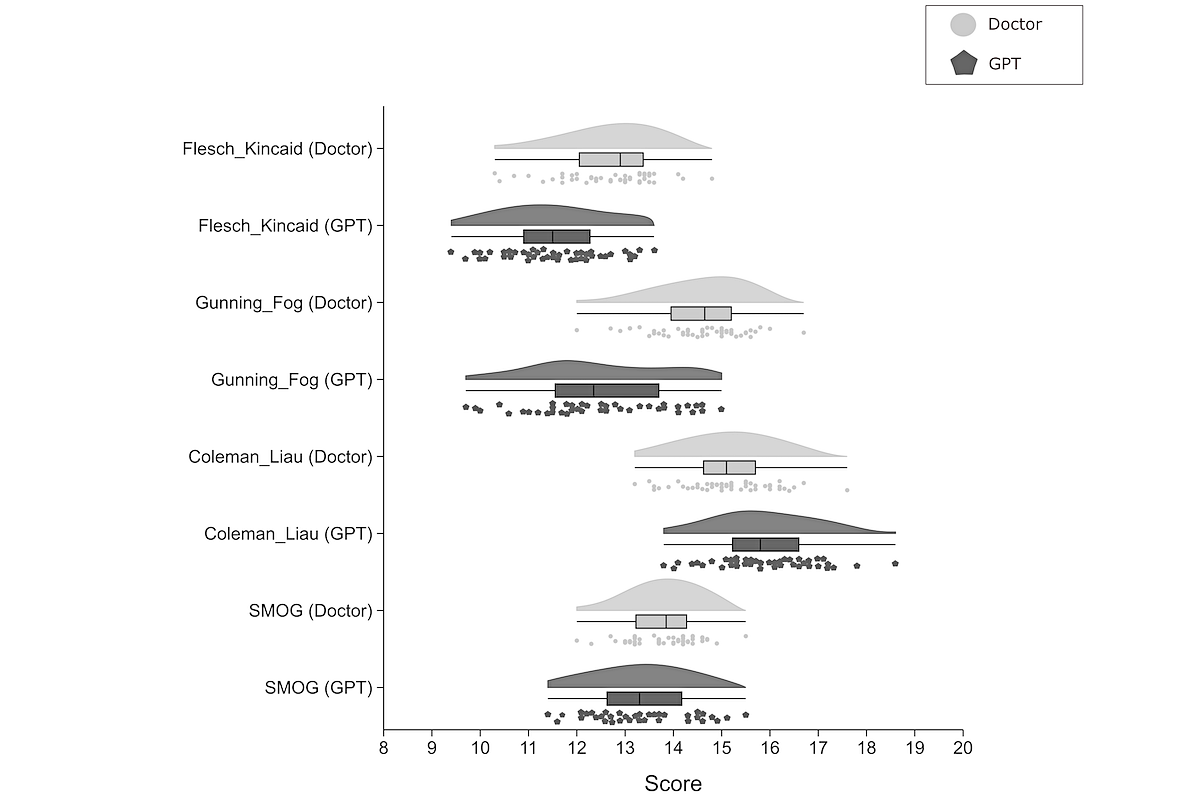
Comparison of readability metrics. Violin plots comparing readability metrics (Flesch-Kincaid, Gunning Fog, Coleman-Liau, and SMOG) between doctor- and GPT-4–generated content, with mean and SD indicated by error bars. SMOG: Simple Measure of Gobbledygook.

A total of 4 validated readability metrics were used to evaluate patient education materials on knee osteoarthritis management generated by clinicians and GPT-4. Significant differences were observed across all metrics (Figure 3). GPT-4–generated content exhibited superior readability on the Flesch-Kincaid grade level (mean 11.56, SD 1.08 for GPT-4 vs mean 12.67, SD 0.95 for clinicians), which assesses the ease of understanding based on word and sentence length, and the Gunning Fog Index (mean 12.47, SD 1.36 for GPT-4 vs mean 14.56, SD 0.93 for clinicians), which estimates the years of formal education needed to comprehend the text. Furthermore, GPT-4 also performed well on the SMOG Index (mean 13.33, SD 1.00 for GPT-4 vs mean 13.81, SD 0.69 for clinicians), which measures the years of education required to understand the material. Clinician-generated materials showed a slight advantage on the Coleman-Liau Index (mean 15.15, SD 0.91 for clinicians vs mean 15.90, SD 1.03 for GPT-4), which considers the number of characters per word and words per sentence. These findings underscore the nuanced strengths of both AI and human-generated materials, with GPT-4 enhancing overall accessibility and quality in patient education.

### Expert Evaluation Results

**Figure 4:**
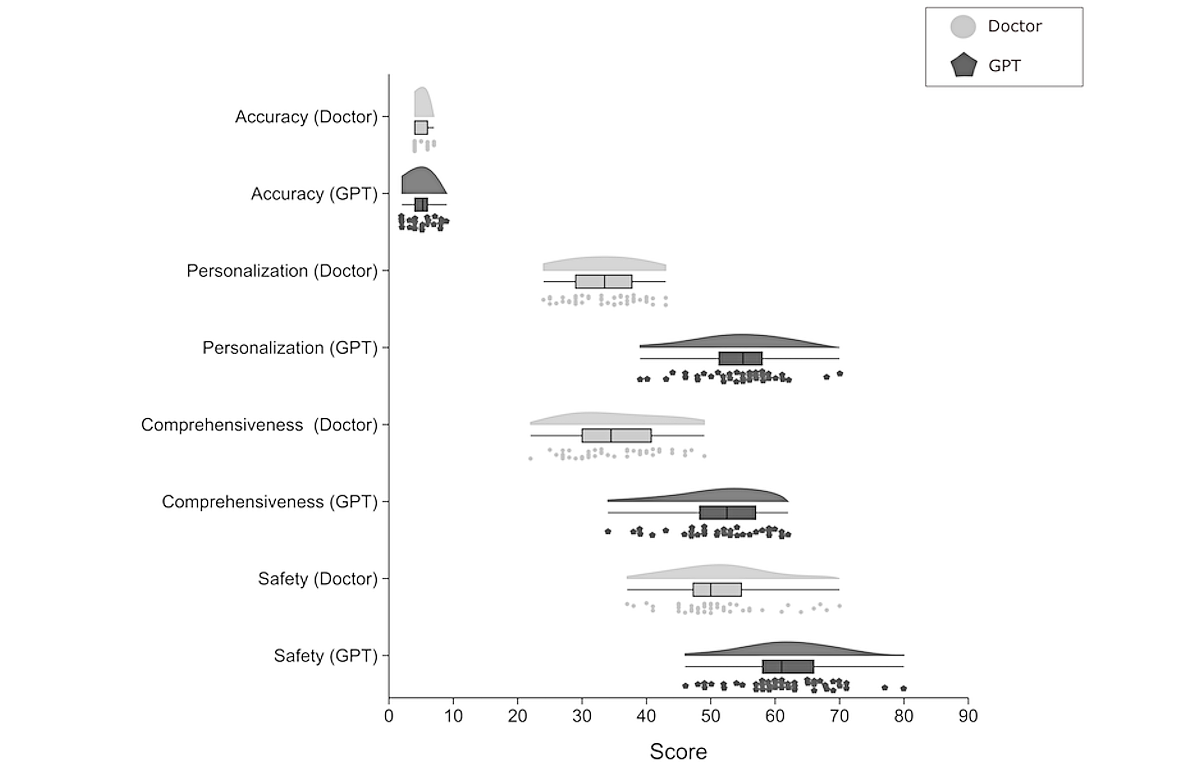
Comparison of accuracy, personality, comprehensiveness, and safety. Violin plots comparing accuracy, personality, and comprehensiveness, with mean and SD shown. Safety uses a box plot to show the median, IQR, and outliers.

GPT-4 demonstrated favorable results across all 4 evaluation dimensions based on expert assessments (Figure 4). For accuracy, clinician-generated materials achieved a mean score of 4.76 (SD 1.10; 4.76/6, 79.3%; *P*=.05), while GPT-4 scored significantly higher at mean 5.31 (SD 1.73; 5.31/6, 88.5%; *P*=.05). In the dimension of personality, GPT-4–generated materials demonstrated a mean score of 54.32 (SD 6.21) out of 100, in stark contrast to the clinician-generated materials, which scored only mean 33.20 (SD 5.40) out of 100 (*P*<.001). Regarding comprehensiveness, GPT-4 again outperformed clinicians, with scores of mean 51.74 (SD 6.47) compared with mean 35.26 (SD 6.66) out of 100 (*P*<.001). Finally, in terms of safety, GPT-4–generated materials received a median score of 61 (IQR 58-66), while clinician-generated content had a median score of 50 (IQR 47-55.25; *P*<.001).

## Discussion

### Principal Findings

Our study reveals that GPT-4 outperformed clinicians in several key aspects of generating personalized self-management guidance for patients with knee OA. In terms of efficiency, GPT-4 demonstrated a clear advantage, producing content significantly faster, which highlights its potential to alleviate clinician workload.

In terms of readability, GPT-4 outperformed clinicians on most validated indices, including the Flesch-Kincaid Grade Level, Gunning Fog Index, and SMOG Index, suggesting generally simpler sentence structure and lower reading difficulty. However, GPT-4 scored slightly higher on the Coleman-Liau Index, which may reflect its tendency to use longer, character-dense words, consistent with its training on formal medical and academic corpora. This lexical precision, while enhancing clinical accuracy, may inadvertently reduce accessibility for patients with limited health literacy. The discrepancy across readability metrics underscores the importance of not only quantifying overall linguistic complexity but also tailoring language to diverse patient populations. These findings highlight the need for future refinement of AI-generated content through adaptive prompt engineering or postprocessing strategies that can optimize readability without compromising informational quality.

On accuracy, GPT-4 performed better, aligning more closely with current medical guidelines, and ensuring that the advice provided is medically reliable. In terms of comprehensiveness, GPT-4 covered a broader range of topics, offering more detailed and thorough information than clinician-generated content. In addition, in personality, GPT-4 excelled by tailoring guidance more effectively to individual patient profiles, considering specific symptoms and lifestyle factors. Finally, GPT-4’s content was rated as safer, consistently adhering to clinical safety standards. These results indicate that GPT-4 has strong potential to improve patient education, particularly in efficiency, accuracy, comprehensiveness, and personality, though readability improvements may be necessary to optimize patient understanding.

### Comparison With Previous Work

The application of LLMs in patient education has evolved through 3 distinct phases, reflecting increasing sophistication and clinical relevance. In the first phase, researchers primarily focused on evaluating the performance of LLMs in answering preset medical questions, often related to specific conditions. Studies like those on urolithiasis and Mohs surgery explored the accuracy, readability, and completeness of LLM-generated responses without direct comparison with human-created content, aiming to assess the baseline capabilities of different models [44,45]. While these studies provided valuable insights into the capabilities of LLMs, they lacked direct comparisons with human-generated content, which is essential for understanding their potential in real-world clinical settings.

In the second phase, LLM-generated outputs were compared with human-generated educational materials or institutional resources. Studies benchmarking LLM responses against traditional patient education materials, such as those related to bariatric surgery and body contouring, highlighted LLMs’ ability to produce content with similar or even superior readability and accuracy [46,47]. These studies marked a significant step forward in validating the use of LLMs for patient education. However, the lack of personalization in the generated content limited their applicability to individual patient care.

Addressing this limitation, the third phase, represented by our research, has pioneered the generation of personalized patient education. By integrating patient-specific factors like medical history and symptomatology, our study on knee osteoarthritis demonstrated that GPT-4 can create tailored educational content that not only matches but often exceeds the quality of clinician-generated materials across dimensions such as accuracy, personalization, and safety. This shift toward highly customized patient education reflects health care’s increasing emphasis on individualized care, especially in managing chronic conditions. With LLMs demonstrating the potential to produce high-quality, personalized educational content, they are poised to enhance patient engagement, self-management, and health outcomes, ultimately paving the way for broader clinical integration and transforming the delivery of patient education [48,49].

### Strengths and Limitations

This study offers several strengths that distinguish it from previous research. First, it leverages the advanced capabilities of GPT-4 to create highly personalized patient education content specifically for knee OA, addressing a significant gap in tailored patient resources. GPT-4’s unique ability to understand and analyze patients’ medical history, symptoms, and lifestyle factors enables it to generate customized educational materials that cater to individual patient needs. In addition, the direct comparison of AI-generated materials with those created by human experts using blinded assessments enhances the rigor and credibility of the findings. By using validated evaluation metrics, the study not only provides a robust assessment of content quality but also sets a new benchmark for future investigations into LLM applications in patient education.

Although the retrospective observational design lacks prospective randomization and may introduce bias, we used several measures to mitigate this. Patient profiles were anonymized and randomly assigned, and both GPT-4 and clinicians generated content under standardized conditions. Evaluations were double-blinded and based on objective, guideline-aligned criteria, with reviewer disagreements resolved through consensus or third-party adjudication. While these controls enhance internal validity, we acknowledge that residual confounding cannot be fully excluded. Future randomized or prospective studies are needed to further validate these findings. Finally, the focus on a common yet impactful condition highlights the practical relevance of the research, facilitating its potential adoption in clinical settings.

However, the study was conducted using data from a single hospital, which may limit the generalizability of the findings to broader populations with different demographic characteristics, cultural backgrounds, or health care infrastructures. As knee osteoarthritis management practices and patient education needs may vary across settings, multicenter studies involving more diverse populations are necessary to validate and extend these results. The lack of assessment of patients’ subjective perceptions and satisfaction hinders understanding of the impact on engagement and adherence. The study did not evaluate long-term effects on patient outcomes, which requires longitudinal investigations. Focusing on a single AI model limits conclusions about other technologies, and comparative studies are needed to identify the most effective approaches. Although evaluations were conducted in a double-blinded manner using standardized criteria, some degree of subjectivity is unavoidable in expert assessments. The involvement of only 2 evaluators may further limit perspective diversity. However, the use of structured scoring frameworks, blinding, and adjudication by consensus or a third reviewer represent significant methodological advancements compared with previous studies. Future research should expand the evaluator pool and incorporate patient-centered assessments to further enhance reliability. Finally, the study did not investigate challenges and barriers to integrating AI-generated content into clinical workflows and its acceptability among health care professionals.

### Future Directions

Future research must rigorously validate the effects of AI-generated content on patient outcomes across diverse populations. Large-scale studies should evaluate both short- and long-term impacts of AI-driven education on patient knowledge, self-management, treatment adherence, patient-reported outcomes, and clinical outcomes [50]. Longitudinal investigations are essential to assess the sustainability and lasting efficacy of AI-assisted interventions.

In addition to expert-based evaluations, future work should incorporate direct patient feedback to assess the perceived usefulness, clarity, and engagement of AI-generated materials. As the ultimate end-users, patients’ perspectives are crucial for determining whether educational content effectively supports self-management and meets individual needs. Mixed methods approach, combining quantitative patient-reported outcomes and qualitative interviews or surveys, may provide a more comprehensive evaluation of AI-assisted education.

This study evaluated only 1 AI model (GPT-4), which may limit the generalizability of findings across different technologies. Future research should include comparative studies involving other LLMs, such as DeepSeek, Claude, or Gemini, to assess variability in medical reasoning, content personalization, and linguistic alignment with local patient populations. Continuous updates and assessments are necessary to address evolving patient needs. In addition, it is crucial to address ethical concerns, such as ensuring transparency, preventing automation bias, avoiding the dissemination of inappropriate or inaccurate content, and protecting patient privacy and data security [51].

Furthermore, the impact of AI-assisted tools on clinical decision-making requires careful exploration. Research should investigate how these technologies influence clinician judgment and whether they enhance or inappropriately replace human decision-making [52]. Future applications of AI in patient education must be guided by clear ethical frameworks, with attention to data privacy, transparency, accountability, and regulatory compliance. Special consideration should be given to the risk of misinformation and the challenge of assigning clinical responsibility when AI-generated guidance is inaccurate, to ensure patient trust and safety.

Future research should also explore collaborative models in which AI-generated educational content is used as a baseline or draft that clinicians can review, modify, and contextualize for individual patients. Such hybrid approaches could combine the efficiency and comprehensiveness of AI with the nuanced judgment, cultural sensitivity, and relational insight of human clinicians. This would not only preserve clinical accountability but also enhance scalability without compromising personalization or safety. Studying how AI-augmented workflows perform in real clinical settings, especially regarding clinician acceptance and patient trust, will be essential to translating these technologies into practice.

### Conclusion

This study demonstrates the potential of GPT-4 in generating personalized patient education content for knee osteoarthritis. The AI-generated content outperformed human expert–created materials in objective efficiency, accuracy, comprehensiveness, personality, and safety. However, clinician-generated content showed a slight advantage in readability on the Coleman-Liau Index. These findings suggest that AI-powered tools have the potential to revolutionize the delivery of tailored health information for chronic conditions. Further research is needed to validate these results in larger populations and real-world clinical settings.

## Appendix

Multimedia Appendix 1 Detailed calculation methodology for weighted consensus approach.

Multimedia Appendix 2 Personalization evaluation method.

Multimedia Appendix 3 Comprehensiveness evaluation method.

Multimedia Appendix 4 Safety evaluation method.

## Abbreviations

AAOS: American Academy of Orthopaedic Surgeons
ACR: American College of Rheumatology
AI: artificial intelligence
ESCEO: European Society for Clinical and Economic Aspects of Osteoporosis, Osteoarthritis and Musculoskeletal Diseases
LLM: large language model
NICE: National Institute for Health and Care Excellence
OA: osteoarthritis
OARSI: Osteoarthritis Research Society International
SMOG: Simple Measure of Gobbledygook
WPM: words per minute

## Data Availability

The datasets generated or analyzed during this study are not publicly available due to patient privacy concerns and institutional data protection policies, but are available from the corresponding author on reasonable request, subject to approval by the institutional ethics committee.
