# Supplementary material for: Comparing Artificial Intelligence–Generated and Clinician-Created Personalized Self-Management Guidance for Patients With Knee Osteoarthritis: Blinded Observational Study
Source: J Med Internet Res. 2025 May 7;27:e67830. doi: 10.2196/67830 (PMC12096024; doi:10.2196/67830)
Supplement: Multimedia Appendix 1 [file jmir_v27i1e67830_app1.docx]

Detailed Calculation Methodology for Weighted Consensus Approach

# Introduction

To assess the accuracy of educational content on knee OA treatment, we employed a weighted consensus approach, synthesizing guidelines from prominent professional societies. These societies include:

- **American College of Rheumatology (ACR)**
- **Osteoarthritis Research Society International (OARSI)**
- **European Society for Clinical and Economic Aspects of Osteoporosis, Osteoarthritis and Musculoskeletal Diseases (ESCEO)**
- **American Academy of Orthopaedic Surgeons (AAOS)**
- **National Institute for Health and Care Excellence (NICE)**

This methodology ensures the alignment of educational materials with the most current evidence-based recommendations.

# Methodology

## Step 1: Categorization and Scoring of Recommendations

Treatment recommendations were categorized and assigned scores based on their endorsement levels:

- **Strongly Recommended**: +2 points
- **Conditionally Recommended**: +1 point
- **Inconclusive**: 0 points
- **Conditionally Recommended Against**: -1 point
- **Strongly Recommended Against**: -2 points

## Step 2: Assigning Consensus Weights

Weights were assigned to each recommendation to reflect the level of agreement among the professional societies. The weighting system is as follows:

- **High Consensus** (4 or more societies in agreement): Weight = 1.0
- **Moderate Consensus** (3 societies in agreement): Weight = 0.75
- **Low Consensus** (2 societies in agreement): Weight = 0.5
- **Minimal Consensus** (1 society in agreement): Weight = 0.25

## Step 3: Calculation of Weighted Scores

The weighted score for each treatment recommendation was calculated using the following formula: Weighted Score = Recommendation Score × Consensus Weight

## Step 4: Example Calculations

**Example 1: Weight Loss**

**Recommendations:**

- ACR: Strongly recommended (+2)
- OARSI: Strongly recommended (+2)
- ESCEO: Strongly recommended (+2)
- AAOS: Strongly recommended (+2)
- NICE: Strongly recommended (+2)

**Consensus Level**: High (5 societies in agreement)

**Consensus Weight**: 1.0

**Weighted Score**: 2×1.0=2

## Step 5: Comprehensive Score Calculation Table

The following table summarizes the consensus levels, weights, and weighted scores for various treatments:

Table 1: Consensus-Based Weighted Scores for Knee Osteoarthritis Treatment Recommendations.

| Treatment | ACR | OARSI | ESCEO | AAOS | NICE | Consensus Level | Consensus Weight | Weighted Score |
| --- | --- | --- | --- | --- | --- | --- | --- | --- |
| Weight Loss | +2 | +2 | +2 | +2 | +2 | High | 1.0 | 2.0 |
| Education Programs | +2 | +2 | +2 | +2 | +2 | High | 1.0 | 2.0 |
| Exercise | +2 | +2 | +2 | +2 | +2 | High | 1.0 | 2.0 |
| Brace | +1 | +1 | +1 | +1 | +1 | High | 1.0 | 1.0 |
| Topical NSAIDs | +2 | +2 | +2 | +2 | +2 | High | 1.0 | 2.0 |
| Oral NSAIDs | +2 | +2 | +2 | +2 | +2 | High | 1.0 | 2.0 |
| Acetaminophen (Paracetamol) | +1 | -1 | -1 | -1 | +1 | Moderate | 0.75 | -0.75 |
| Tramadol | +1 | +1 | +1 | +1 | +1 | High | 1.0 | 1.0 |
| Duloxetine | +1 | +1 | +1 | +1 | +1 | High | 1.0 | 1.0 |
| Intra-articular Corticosteroids | +1 | +1 | +2 | +1 | +2 | High | 1.0 | 1.4 |
| Intra-articular Hyaluronic Acid | -1 | -1 | -1 | -1 | -1 | High | 1.0 | -1.0 |
| Platelet-rich Plasma | -2 | -2 | -2 | -2 | -2 | High | 1.0 | -2.0 |
| Stem Cell Injection | -2 | -2 | -2 | -2 | -2 | High | 1.0 | -2.0 |
| Intra-articular Prolotherapy | -2 | -2 | -2 | -2 | -2 | High | 1.0 | -2.0 |

## Step 6: Aggregation of Scores

The overall accuracy score for the educational content was determined by summing the weighted scores for all treatments mentioned. Higher scores indicate better alignment with consensus guidelines, while lower or negative scores highlight areas for potential revision.

# Conclusion

The weighted consensus approach provides a rigorous and objective methodology for evaluating the accuracy of educational content on knee OA treatment, with a 6.0-point score defined as the standard for excellence. By considering the varying levels of agreement among professional societies, this approach ensures that the content reflects the most current and widely accepted clinical guidelines. This enhances the reliability and clinical relevance of the educational materials provided to patients, ultimately improving patient care and outcomes.
