# Supplementary material for: Comparing Artificial Intelligence–Generated and Clinician-Created Personalized Self-Management Guidance for Patients With Knee Osteoarthritis: Blinded Observational Study
Source: J Med Internet Res. 2025 May 7;27:e67830. doi: 10.2196/67830 (PMC12096024; doi:10.2196/67830)
Supplement: Multimedia Appendix 2 [file jmir_v27i1e67830_app2.docx]

Personalization Evaluation Method

# Introduction

To assess the degree of personalization of the content of the knee osteoarthritis treatment communication, a systematic scoring method was used to ensure that the content could be personalized according to the patient's specific characteristics, symptoms, stage of the disease, lifestyle habits and past medical history. The steps are described below:

# Personalization Scoring System

## Relevance (30 points)

| **Evaluation Item** | **Evaluation Standard** | **Specific Description** | **Score (0-15 points)** |
| --- | --- | --- | --- |
| **Symptom Relevance** | Highly relevant (13-15pts) | Detailed description of the patient's specific symptoms |  |
|  | Mostly relevant (9-12 pts) | Mostly describes the patient's symptoms, some details missing |  |
|  | Partially relevant (5-8 pts) | Describes some symptoms, overall relevance is weak |  |
|  | Low relevance (1-4 pts) | Involves few symptoms |  |
|  | Almost no relevance (0 pts) | Almost no relevance to the patient's symptoms |  |
| **Disease Stage Relevance** | Highly relevant (13-15pts) | Detailed description of the patient's disease stage |  |
|  | Mostly relevant (9-12 pts) | Mostly describes the disease stage, some details missing |  |
|  | Partially relevant (5-8 pts) | Describes some disease stages, overall relevance is weak |  |
|  | Low relevance (1-4 pts) | Involves few disease stages |  |
|  | Almost no relevance (0 pts) | Almost no relevance to the patient's disease stage |  |

## Individual Relevance (40 points)

| **Evaluation Item** | **Evaluation Standard** | **Specific Description** | **Score  (0-10 points)** |
| --- | --- | --- | --- |
| **Customization to Individual Characteristics** | Highly personalized (13-15 pts) | Fully customized to the patient's individual characteristics |  |
|  | Mostly personalized (9-12 pts) | Mostly personalized content, some characteristics missing |  |
|  | Partially personalized (5-8 pts) | Partially personalized content, many aspects not considered |  |
|  | Low personalization (1-4 pts) | Only considers few individual characteristics |  |
|  | Almost no personalization (0 pts) | Almost no consideration of the patient's individual characteristics |  |
| **Consideration of Lifestyle Habits** | Highly considered (9-10 pts) | Fully considers the patient's lifestyle habits |  |
|  | Mostly considered (7-8 pts) | Mostly considers lifestyle habits, some details missing |  |
|  | Partially considered (5-6 pts) | Partially considers lifestyle habits, overall consideration is weak |  |
|  | Low consideration (3-4 pts) | Involves few lifestyle habits |  |
|  | Almost no consideration (0-2 pts) | Almost no consideration of the patient's lifestyle habits |  |
| **Integration of Medical History** | Highly integrated (9-10 pts) | Fully integrates the patient's medical history and current medications |  |
|  | Mostly integrated (7-8 pts) | Mostly integrates medical history and medications, some details missing |  |
|  | Partially integrated (5-6 pts) | Partially integrates medical history and medications, overall integration is weak |  |
|  | Low integration (3-4 pts) | Involves few aspects of medical history and medications |  |
|  | Almost no integration (0-2 pts) | Almost no integration of the patient's medical history and medications |  |

## Detail Level (30 points)

| **Evaluation Item** | **Evaluation Standard** | **Specific Description** | **Score (0-15 points)** |
| --- | --- | --- | --- |
| **Information Depth** | Very detailed (13-15 pts) | Provides rich details and in-depth analysis |  |
|  | Detailed (9-12 pts) | Detailed content, but some aspects could be deeper |  |
|  | Some detail (5-8 pts) | Contains some details, but overall depth is insufficient |  |
|  | Rough (5-8 pts) | Rough content, lacks in-depth analysis |  |
|  | Very rough (1-4 pts) | Very rough content, lacks basic details |  |
|  | No detail (0 pts) | Very rough and lacks necessary information. |  |
| **Specific Suggestions** | Very practical (13-15 pts) | Suggestions are very specific, practical, and highly tailored to the patient's condition |  |
|  | Practical (9-12 pts) | Suggestions are quite specific, but some aspects are vague |  |
|  | Some practicality (5-8 pts) | Suggestions have some practicality, but overall targeting is weak |  |
|  | Vague (1-4 pts) | Suggestions are vague, lacking practicality |  |
|  | Almost no practicality (0 pts) | Suggestions are very rough, almost no practical value |  |

# Overall Score Calculation

The scores from each category are summed up to get a total score out of 100 points. Each evaluation criterion is rated individually to provide a detailed breakdown of the personalization level.
