# Supplementary material for: Comparing Artificial Intelligence–Generated and Clinician-Created Personalized Self-Management Guidance for Patients With Knee Osteoarthritis: Blinded Observational Study
Source: J Med Internet Res. 2025 May 7;27:e67830. doi: 10.2196/67830 (PMC12096024; doi:10.2196/67830)
Supplement: Multimedia Appendix 3 [file jmir_v27i1e67830_app3.docx]

Comprehensiveness Evaluation Method

# Introduction

To ensure the comprehensiveness of patient education content generated by both human experts and large language models, we have developed a thorough evaluation method. This method ensures that the content sufficiently covers all necessary topics related to patient care and provides essential information for patients to manage their conditions effectively. Below are the detailed steps for the comprehensiveness evaluation:

# Safety and comprehensiveness Evaluation Criteria

The scoring standards are based on one key dimension: Comprehensiveness (whether the content thoroughly covers all necessary aspects). Each evaluation aspect has a maximum score of 20 points, totaling 100 points across five categories (Medication Treatment, Non-Medication Treatment, Lifestyle Advice, Psychological Support, and Disease Management).

# Scoring System

| **Evaluation Item** | **Evaluation Standard** | **Specific Description** | **Score (0-20 points)** |
| --- | --- | --- | --- |
| **Medication Treatment** | Completely covered (17-20 pts) | Covers all key aspects of medication, including interactions and contraindications. |  |
|  | Mostly covered (13-16 pts) | Covers most aspects, with minor omissions. |  |
|  | Partially covered (9-12 pts) | Mentions a few key points but misses several important details. |  |
|  | Rarely covered (5-8 pts) | Provides minimal or incomplete information. |  |
|  | Not covered (0-4 pts) | Does not cover medication use. |  |
| **Non-Medication Treatment** | Completely covered (17-20 pts) | Fully covers all relevant non-medication treatments. |  |
|  | Mostly covered (13-16 pts) | Covers most relevant treatments, with some omissions. |  |
|  | Partially covered (9-12 pts) | Mentions a few treatments but omits significant ones. |  |
|  | Rarely covered (5-8 pts) | Minimal information on non-medication treatments. |  |
|  | Not covered (0-4 pts) | Does not cover non-medication treatments. |  |
| **Lifestyle Advice** | Completely covered (17-20 pts) | Comprehensive lifestyle advice, including diet, exercise, and sleep. |  |
|  | Mostly covered (13-16 pts) | Covers most lifestyle advice, with minor omissions. |  |
|  | Partially covered (9-12 pts) | Provides minimal lifestyle advice. |  |
|  | Rarely covered (5-8 pts) | Barely mentions lifestyle adjustments. |  |
|  | Not covered (0-4 pts) | Does not address lifestyle advice. |  |
| **Psychological Support** | Completely covered (17-20 pts) | Covers psychological support and mental health strategies. |  |
|  | Mostly covered (13-16 pts) | Covers most aspects with some details missing. |  |
|  | Partially covered (9-12 pts) | Mentions minimal psychological support. |  |
|  | Rarely covered (5-8 pts) | Barely covers psychological support. |  |
|  | Not covered (0-4 pts) | Does not address psychological support. |  |
| **Disease Management** | Completely covered (17-20 pts) | Thorough coverage of disease and comorbidity management. |  |
|  | Mostly covered (13-16 pts) | Covers most disease management aspects. |  |
|  | Partially covered (9-12 pts) | Minimal coverage of disease management. |  |
|  | Rarely covered (5-8 pts) | Barely mentions disease management. |  |
|  | Not covered (0-4 pts) | Does not address disease management. |  |

# Conclusion

By using this systematic evaluation method, we can thoroughly review and quantify the comprehensiveness of the educational content, ensuring that it covers all critical aspects required for patient education and disease management. This approach guarantees that each aspect of the recommendations is sufficiently addressed, providing patients with well-rounded, informative content to support their care.
